# Gender biases in the training methods of affective computing: Redesign and validation of the Self-Assessment Manikin in measuring emotions *via* audiovisual clips

**DOI:** 10.3389/fpsyg.2022.955530

**Published:** 2022-10-20

**Authors:** Clara Sainz-de-Baranda Andujar, Laura Gutiérrez-Martín, José Ángel Miranda-Calero, Marian Blanco-Ruiz, Celia López-Ongil

**Affiliations:** ^1^Department of Communication and Media Studies, Universidad Carlos III de Madrid, Getafe, Madrid, Spain; ^2^Institute on Gender Studies, Universidad Carlos III de Madrid, Getafe, Madrid, Spain; ^3^Department of Electronic Technology, Universidad Carlos III de Madrid, Leganés, Spain; ^4^Department of Audiovisual Communication and Advertising, Universidad Rey Juan Carlos, Fuenlabrada, Spain

**Keywords:** Self-Assessment Manikin, gender, emotion, affective space, pleasure-arousal-dominance, affective computing, artificial intelligence

## Abstract

Audiovisual communication is greatly contributing to the emerging research field of affective computing. The use of audiovisual stimuli within immersive virtual reality environments is providing very intense emotional reactions, which provoke spontaneous physical and physiological changes that can be assimilated into real responses. In order to ensure high-quality recognition, the artificial intelligence (AI) system must be trained with adequate data sets, including not only those gathered by smart sensors but also the tags related to the elicited emotion. Currently, there are very few techniques available for the labeling of emotions. Among them, the Self-Assessment Manikin (SAM) devised by Lang is one of the most popular. This study shows experimentally that the graphic proposal for the original SAM labelling system, as devised by Lang, is not neutral to gender and contains gender biases in its design and representation. Therefore, a new graphic design has been proposed and tested according to the guidelines of expert judges. The results of the experiment show an overall improvement in the labeling of emotions in the pleasure–arousal–dominance (PAD) affective space, particularly, for women. This research proves the relevance of applying the gender perspective in the validation of tools used throughout the years.

## Introduction

The last decades have witnessed a growing interest in the multisensorial and multimodal aspects of science and technology, the integration of the measurement of emotion through the use of smart sensors being one of the emerging research lines in fields such as communication, engineering, and psychology among others. Affective computing is based on the study, analysis, and interpretation of human emotional reactions by means of artificial intelligence (AI; Picard, 1995; Picard et al., 2001), which requires the usage of complete databases where not only the measurements from different sensors are compiled rigorously but also the tags of the experimented emotions. These tags can be unconstrained or previously predefined. The predefined ones can be discrete—chosen from a finite, predefined set of emotions—or continuous, within a predefined affective space, such as the tridimensional pleasure–arousal–dominance (PAD) space (Fontaine et al., 2016), where the experimented emotion is represented *via* numerical values on a Likert scale in the dimensions of pleasure, arousal and dominance. In any case, the tags must always be gathered while the different emotions are being elicited in volunteers *via* various external stimuli.

The most used scientific databases for the study of emotions—MANHOB (Soleymani et al., 2012) and DEAP (Koelstra et al., 2012)—use the Self-Assessment Manikin (SAM) designed by Lang (1980) and Hodes et al. (1985) in the 1980s, first as a computerised, interactive graphical interface tool, although a manual version of it was later made. In fact, this non-verbal pictorial assessment technique has generally been adopted for mapping emotions in a three-dimensional space (PAD), according to the levels of pleasure (P), arousal (A), and dominance (D) every emotion draws out of the person.

The SAM technique has been consolidated throughout the years in the scientific community as a globally reliable system to classify emotions. It provides a well-defined measure with strong psychometric properties (Lang, 1980; Bradley and Lang, 1994; Leen-Feldner et al., 2008; Olatunji et al., 2009; Soares et al., 2013; Bilsky et al., 2018). For example, in their study, Zaki and Ochsner (2015) confirm that the manikins allow people to express their emotional reactions beyond linguistic barriers or discrete labels, leveraging their empathy with the figures’ expressions when observing and contemplating the image or drawing.

The SAM system provides three independent scales—PAD—associated with the emotional response to external stimuli. Each scale contains five similar figures with different expressions:

- The first scale (valence/pleasure) ranges from positive sensations to negative feelings. The farthest figure on the left shows a smile, while the one farthest to the right displays a worried/sad expression.
- The second scale (arousal/excitement) measures from the highest states of excitement to calmness. The farthest figure on the left seems ready to explode, while the one on the opposite side looks calm or asleep.
- The third scale, related to dominance, corresponds to the ability to control the intensity of the emotion experimented by the subject (Verma and Tiwary, 2015); it presents a small human figure in the square, growing from left to right, where it can be seen outside of the square because of its size.

Through these images, the person can mark any figure or space between two figures with an “x” to indicate the closest emotion to the elicited one.

For the most part, SAMs have suffered variations in the sequential order of the figures in the scales of valence and arousal, being displayed from negative to positive feelings in the case of valence and from calmness to excitement in arousal (Koelstra et al., 2012; Miranda-Correa et al., 2018). This variation in the figures’ sequential order must be considered for future comparisons with results from different research papers published.

The manikins have also suffered aesthetical modifications in the figures’ design (Koelstra et al., 2012; Miranda-Correa et al., 2018), up to the point of proposing the use of avatars instead of manikins (Sonderegger et al., 2016). Nonetheless, these modifications have not been validated through experimental research to the best of our knowledge, nor have they considered sociocultural or gender biases.

In this context, keeping in mind that one of the main objectives of this study is the validation of aesthetic modifications of the manikins, cultural and gender biases should be taken into consideration in the same way as the contents of video clips used to cause emotional reactions in order to generate audiovisual databases—the UC3M4Safety database for Spain (Blanco-Ruiz et al., 2021a,b) or Emotional Film for Asian culture (Deng et al., 2017). Gender and cultural differences have also been confirmed (Gantiva et al., 2011; Moltó et al., 2013) in the International Affective Picture System (IAPS; Lang et al., 2008), which includes over 1,000 pictures that represent a set of normative emotional stimuli for experimental research about attention and emotions.

The identification with human-like figures is a key concept in understanding and explaining the processes and effects that the stimuli provoke in the subjects while the experiments are being conducted. Through the figures, many emotions felt during direct encounters in personal experiences are recalled, activating what is known as autobiographical memory (Cohen, 2001; Sainz-de-Baranda et al., 2021b).

The different experiments in emotion recognition have detected that, in addition to individual differences in empathising with others (Lockwood et al., 2017; Israelashvili et al., 2019; Blanco-Ruiz et al., 2020; Sainz-de-Baranda et al., 2021a), there are also cultural, linguistic, sexual and age differences (Hagemann et al., 1999; Trommsdorff et al., 2016; Di Girolamo et al., 2019; Ge et al., 2019; Grégoire and Greening, 2020) that should be addressed and adapted so that every subject can reach a greater empathy with the audiovisual speeches being studied. In this sense, recent studies from feminist technoscience studies have highlighted that digital technologies and AI have biases in terms of gender, sex, job, class, ethnicity, and (dis)ability among others (Sumartojo et al., 2016; Hicks, 2017; Dunbar-Hester, 2019; Thaler, 2022).

Gender^1^ analysis of the world around us, and thus of technology, shows that from its design to its operation, it is not gender neutral (Haraway, 1988; Harding, 1991; Wajcman, 2006; Zafra, 2011). Examples, such as the design of autonomous cars with a gender perspective to correct inequalities in the design of the traditional belt (Saleh et al., 2022), differences in cardiovascular rehabilitation (Kentner and Grace, 2017) or the John–Jennifer effect (Moss-Racussin et al., 2012), are evidence of the need for this shift towards gender sensitivity. However, this perspective must be complemented by the intersectional perspective (Crenshaw, 1991). Recent studies on the effects of AI algorithms, such as the studies by Buolamwini and Gebru (2018), Cirillo et al. (2020), Noble (2018), and Nurock (2020) among others, point out that not only gender biases are reproduced but also those of race, class, or age.

In Europe, the European Commission (2020) has incorporated the gender perspective and the intersectional perspective into research and innovation content in the Horizon Europe framework programme, with AI being one of the key areas. Examples of this line of work include projects such as VITAPATCH in Austria, which are developing a multifunctional data patch for vital and movement monitoring in everyday environments, where its researchers are integrating knowledge on feminist technoscience into the technology design process. In the case of Spain, the EMPATÍA-CM project is working to generate automatic detection mechanisms to protect victims of gender-based violence in situations of danger, and from its beginnings, it has incorporated the gender and victim perspective into its development. As Tannenbaum et al. (2019) point out; taking a gender-sensitive view improves science and technology.

In this context, and considering that one of the main objectives of this work is the validation of aesthetic modifications of the manikins, cultural and gender biases should be taken into consideration.

## Materials and methods

The initial hypothesis of this research was that the tools designed and traditionally used to measure emotions, and therefore train the intelligent systems used in affective computing, were not gender neutral. For this reason, they required a methodological revision from the gender studies perspective to produce a more equal, inclusive, and diverse science.

The aim of this study was to validate aesthetic modifications to the SAMs that serve in tagging emotions within the PAD space. This question arose when the multidisciplinary UC3M4Safety team raised the need to generate an audiovisual database—the UC3M4Safety database (Blanco-Ruiz et al., 2021a,b)—to elicit emotions through audiovisual stimuli and launch an intelligent system with the ability to determine the emotional state of a person (San-Segundo et al., 2021) known as Bindi (Miranda et al., 2021). In this sense, this work focused on analysing possible gender biases in the labelling system and thus avoiding their effects in emotion recognition. It is important to note that the labelling system conditioned the resulting intelligent system because the latter is based on supervised learning.

In this section, the different aspects of the methodology followed by this research are detailed (Ortega-Toro et al., 2008). First, the protocol, the participants, and the design of the different experiments conducted are explained and, finally, the instrument of reference is shown (Supplementary material).

### Protocol

**Figure 1:**
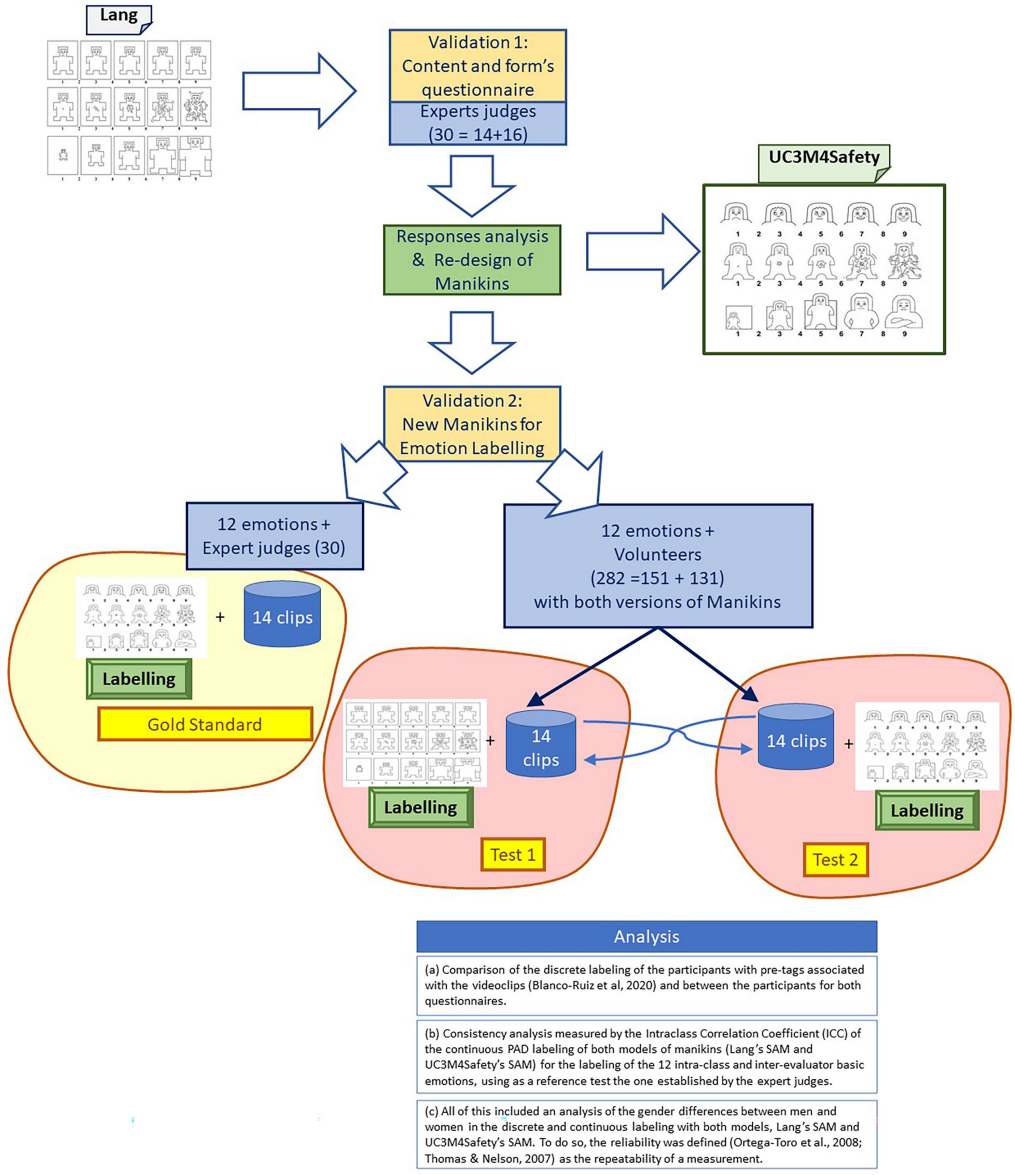
Stages and procedures involved in methodology.

In the design of questionnaires for emotional self-labelling, we have used a stepping stone of those questionnaires that are currently used in scientific databases devoted to studying emotions and that use audiovisual stimuli of different natures to elicit emotions: FilmStim (Schaefer et al., 2010), MANHOB (Soleymani et al., 2012), DEAP (Koelstra et al., 2012), and the Emotional Film database for Asian culture (Deng et al., 2017). These are among the most used and referenced ones. All of them use the SAM tool as the emotion labelling procedure in the PAD space. It is worth noting that, despite its use in these and other publications within the field, more research on the PAD model is still needed to conceive it as a solid and proved emotional dimensional model (Bakker et al., 2014). Thus, this work claims to deepen this kind of research and deals specifically with the gender bias problem within this field. To this end, the protocol followed is based on the three following phases (Figure 1):

- The first phase was aimed at acquiring the validity of the content and the form of the survey (Table 1). To this end, the questionnaire that included the SAMs with the original aesthetic designed by Lang (1980) was sent to a group of expert judges (16 women and 14 men).
- The second phase consisted of the interpretation of each of the expert judges’ answers, after which the original aesthetic of the manikins was redesigned (Table 2).
- In the third phase, a two-step experiment was designed to confirm or discard the improvement in labelling between Lang’s SAMs and those designed by the UC3M4Safety team (UC3M4Safety’s SAMs), namely:
  1. Asking the expert judges to label 12 basic emotions—described in the “Instrument” section, Table 3. This labeling has been used as the reference test (gold standard) in order to compare them with the labels provided by the sample.
  2. Conducting an experiment where a sample of persons, divided into two groups, use both models of the SAMs under comparison to label a set of audiovisual stimuli (with emotional content); each group uses the two models of the SAMs in a different order to avoid biases.

**Table 1:** Quantitative assessment issued by the expert judges about the Self-Assessment Manikins (Lang’s vs. UC3M4Safety’s).

**Items**	**Lang’s SAMs**			**UC3M4Safety’s SAMs**		
	**Content**	**Form**	**Mean**	**Content**	**Form**	**Mean**
Valence	0.830	0.722	0.776	0.983	0.963	0.973
Arousal	0.873	0.827	0.850	0.980	0.990	0.985
Dominance	0.867	0.643	0.755	0.993	0.980	0.987

Aiken’s V coefficient for content and form validity ranges from 0 to 1.

Degree of belonging to the subject study (content). The extent to which each of the questionnaire’s items was supposed to take part in the instrument was registered. To achieve this, the expert judges indicated in a scale from 0 to 10 the degree of belonging of the item to the instrument (0 = not relevant, 10 = highly relevant).

Degree of accuracy and adequacy (form). The extent to which each of the questionnaire’s items accurately defined its objective was registered. Likewise, the expert judges indicated in a scale from 0 to 10, the degree of accuracy in the definition and wording of the instrument (0 = inadequate, 10 = highly adequate).

**Table 2:** Qualitative assessment issued by the expert judges about Self-Assessment Mankins of Lang (1985).

**Judge**	**Sex**	**Age**	**Specialty**	**Assessment**
1	Woman	52	Clinical Psychology	I think the SAMs are accurate because the body reflects the degree of arousal, and it is clearer than the face. However, I would make a change so that people can empathise better. The faces of the SAMs are very small in relation to the body, and the face should be highlighted more so that it reflects (un)happiness better and more visibly. I would remove the titles of valence, arousal and dominance.
2	Woman	57	Gender Studies/ Sociology	The images are very explicit, as a reflection from where the emotion is felt, but they are masculinised (more in the MANHOB); I would change the dummies or shapes.
3	Man	57	Clinical Psychology	The titles of valence, arousal, and dominance create confusion. Even though Lang’s dummies are clear and simple, I would make them more neutral, with curves.
4	Man	45	Clinical Psychology	No comments.
5	Man	44	Psychology	No comments.
6	Woman	51	Sociology	I would remove the first “arousal” statement in the text. Arousal, nervousness and activation are easily identifiable in the manikins. I like the order from lower to higher in the shapes. Even so, I feel displeased by the drawings; I will not relate to them, especially the ones from 5 to 9.
7	Man	41	Clinical Psychology	They are fine. Consider having a male or female dummy according to the person’s sex.
8	Man	45	Clinical Psychology	I am not convinced by the dummies. Perhaps a dummy should be made for men and another for women.
9	Woman	40	Clinical Psychology	There are words that may lead to an error (valence, arousal, and dominance). The drawings are good and illustrative, but a bit masculine. I would make more feminised dummies.
10	Woman	42	Clinical Psychology	The shape of the dummy is very masculine. Please improve the facial expressions. [Make] the facial expression less aggressive. Eliminate the word “dominance” and replace it with another; “dominant” refers to the dominance of a third person.
11	Woman	42	Clinical Psychology	The term “dominance” should be changed.
12	Woman	51	Communication	It is not clear when the arousal and valence categories are used; I would eliminate them. The same logic applies to dominance; I would replace it with “control.” I would change the dummies so that they are more neutral.
13	Woman	31	Psychology	I would modify the manikins and highlight the faces more. Additionally, they are a bit masculine, especially when they are shown to women. I prefer the low-to-high sequential order.
14	Man	51	Communication	Consider having a male or female dummy according to the person’s sex.
15	Woman	38	Psychology/Neuroscience	Seek less robotic and masculine facial expressions. They are not relatable.
16	Woman	42	Psychology/Neuroscience	I would prefer a more neutral set of dummies. Eliminate “arousal,” “valence,” and “dominance” because they are misleading. Substitute “dominion” with “control.”
17	Woman	50	Gender Studies/ Sociology	Arousal has a sexual connotation; it would be better to change that word. Consider having a male or female dummy according to the sex of the participant. I would remove the title “valence” and leave “How do you feel?”
18	Man	57	Publicity	Seek an alternative to the manikins’ faces, something more neutral or feminine.
19	Man	45	Clinical Psychology	It is not clear. I do not like the manikins; they are not relatable.
20	Man	44	Psychology	I would pursue a more neutral dummy. Consider using emojis.
21	Woman	51	Publicity	The images are very explicit, reflecting where the emotion is felt, but the SAMs are masculinised in all the squares.
22	Man	41	Clinical Psychology	Consider having a male or female dummy according to the sex of the person, or make something more neutral.
23	Man	45	Publicity	I would change the manikins, highlighting the faces more and making them less masculine.
24	Woman	40	Publicity	No comments.
25	Man	42	Clinical Psychology	The drawings are very good, and the graphics are illustrative, but I am not convinced by the fact that they are so masculinised.
26	Man	45	Clinical Psychology	No comments.
27	Woman	51	Communication	No comments.
28	Woman	47	Communication	Perhaps the SAMs could be redesigned for men and women specifically.
29	Man	51	Publicity	Design more neutral manikins.
30	Woman	38	Psychology/Neuroscience	Pursue less masculine facial expressions.

**Table 3:** Classification of discrete emotions in the UC3M4Safety database (Blanco-Ruiz et al., 2021a,b).

Joy (Happiness, satisfaction)	Sadness (distress, sorrow)
Surprise (amazement, amusement)	Contempt (indifference, antipathy)
Hope (trust, safety, and faith)	Fear (distrust, anguish, and anxiety)
Attraction (desire, interest)	Disgust (repugnance, aversion)
Tenderness (Gratitude, satisfaction)	Anger (annoyance, ire, irritation, fury, and rage)
Calm (tranquillity, peace)	Tedium (boredom, weariness)

Self-elaborated.

The results validate both test A (Lang) and test B (UC3M4Safety) with the gold standard.

### Sample

**Figure 2:**
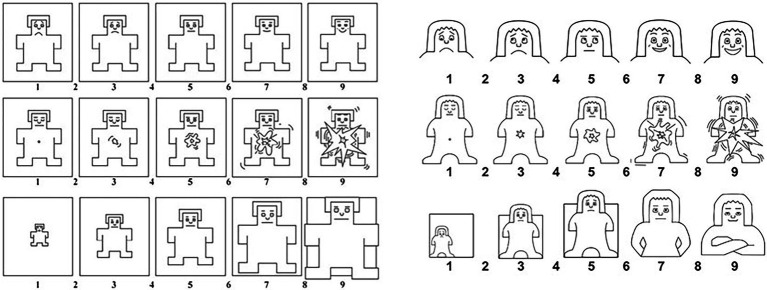
Models of the Self-Assessment Manikins proposed by Lang/UC3M4Safety.

In the three stages of the protocol, 30 expert judges—16 women and 14 men—took part in this experiment, out of which 16 were female researchers in the fields of communication, publicity, sociology, psychology, and gender studies, and the remaining 14 were male clinical psychologists and neuropsychologists. All of them had wide professional experience (over 6 years) and knowledge of gender perspective due to their profession or tuition. The age of the participants ranged between 38 and 57 years old. All participants were Spanish speakers from the Ibero-American countries. These expert judges were asked to assess the validity of the content and the form of both manikin models (SAM Lang/SAM UC3M4Safety, Figure 2), as well as to label 12 discrete emotions selected with the SAM UC3M4Safety model (as described in the “Instrument” section, Table 3). This labeling was used as a reference test in the last phase of the experiment. The sampling method was non-probabilistic, snowball sampling. The expert judges participated voluntarily. They were informed in advance of the aims of the study and the treatment of the data collected, and they had as much time as they considered necessary.

In the third phase (2), in order to obtain the information about the labelling comparison of both manikin models (Figure 2), a sample of 282 people (151 women and 131 men) was recruited *via* an intentional sampling among students and professors in advertising and marketing studies (bachelor’s and master’s degrees in 2020/21 and 2021/22 academic years) from universities in the region of Madrid. The sample was between 20 and 52 (32.14 ± 9.09) years old. Previously, all were informed of the study’s purpose and the treatment of the data collected. Only those who voluntarily agreed to participate in the experiment were recruited.

Before the online questionnaires were disseminated (through the Google Form platform), all participants received a lesson on measuring emotions through audiovisual stimuli and the different variables included in the SAM labelling procedure (valence, arousal, and dominance).

Afterward, those who agreed to participate voluntarily completed the questionnaire. All people were Spanish-speaking or fluent in Spanish (a prerequisite for evaluating the video clips that formed part of the sample).

The survey was conducted individually *via* each person’s personal electronic devices. It was distributed during the months of October 2021 to February 2022. The average response time was 30 min.

### Design

As indicated in the procedure description, section “Protocol”, the study of the validity of the questionnaires that included the SAMs was conducted in the first phase, taking the “validity of the content” as the degree to which a test represented adequately its mission or objective (Wiersma, 2001; Thomas and Nelson, 2007; Ortega-Toro et al., 2008).

In order to reach optimal levels of content validity in the questionnaire designed for the collection of discrete tags (discrete emotions) and continuous tags (PAD space represented by SAM), the technique of the expert judges (Pedrosa et al., 2013) was used. To that end, these judges were asked to assess different aspects of the initial information, the measurement scale, and the questionnaire items and to perform a global assessment of each (Wiersma, 2001; Ortega-Toro et al., 2008). This process was carried out in two phases: first, Lang’s SAMs were assessed, and then UC3M4Safety’s SAMs, following the guidelines obtained in the first phase. Regarding each item of the instrument, the judges were asked to indicate the:

1. Degree of belonging to the subject study (content). The extent to which each item of the questionnaire was supposed to take part in the instrument was registered. To achieve this, the expert judges indicated in a scale from 0 to 10 the degree of belonging of the item to the instrument (0 = not relevant, 10 = highly relevant).
2. Degree of accuracy and adequacy (form). The extent to which each of the questionnaire’s items accurately defined its objective was registered. Likewise, the expert judges indicated in a scale from 0 to 10 the degree of accuracy in the definition and wording of the instrument (0 = inadequate, 10 = highly adequate).
3. Global assessment of each item.

In the third phase, as described in the “Protocol” section, the experiment was designed to measure the validity of the labelling of the new manikins (SAM UC3M4Safety) and compare them with Lang’s SAM. The experiment was proposed and designed to check if the new manikins (SAM UC3M4Safety) improved the labelling procedure, leveraging the results for both genders and bringing them closer to the “golden” labels. The spirit of the experiment stemmed from the proposal by Ortega-Toro et al. (2008). The phases of the experiment were:

1. First of all, the expert judges established the references for the 12 basic emotions in the PAD tridimensional space (valence, arousal, and dominance). These basic emotions were tedium, joy, disgust, attraction, contempt, hope, tenderness, anger, fear, surprise, calm, and sadness, as described in the “Instrument” section (Table 3). Emotions were balanced between positive and negative emotions.
2. Second of all, the experiment was designed so that every participant performed two tests using Lang’s SAM with a change in the sequential order as proposed by MANHOB (Soleymani et al., 2012) and DEAP (Koelstra et al., 2012) and recommended by the experts. Additionally, the UC3M4Safety SAMs were designed following the recommendations of the experts. The participants assessed each video in the three PAD dimensions, marking an “x” on each of the five figures or in any of the spaces between them, resulting in a score ranging from 1 (minimal pleasure, minimal activation, and minimal control) to 9 (maximum pleasure, maximum activation, and maximum control) per dimension.

Both questionnaires were completed by 282 participants (151 women and 131 men). The measurements were separated in time by 1 week, and they were performed in practically identical circumstances (Baumgartner, 2000).

Twelve video clips were assessed in each questionnaire, which had been previously tagged with the 12 selected basic emotions (Blanco-Ruiz et al., 2020). The videos used, one for each target emotion, were extracted from the UC3M4Safety database.^2^ Two groups were created to alternate the original manikins with the new designs in order to avoid labelling biases due to the sequential order in which they were presented.

3. Finally, the responses of the participants were analysed in three aspects:

- Comparison of the discrete labeling of the participants with pre-tags associated with the video clips (Blanco-Ruiz et al., 2020) and between the participants for both questionnaires
- Consistency analysis measured by the intraclass correlation coefficient (ICC) of the continuous PAD labelling of both models of manikins (Lang’s SAM and UC3M4Safety’s SAM) for the labelling of the 12 intraclass and interevaluator basic emotions, using as a reference test the one established by the expert judges
- All of this included an analysis of the gender differences between men and women in the discrete and continuous labeling with both models, Lang’s SAM and UC3M4Safety’s SAM. To do so, reliability was defined (Thomas and Nelson, 2007; Ortega-Toro et al., 2008) as the repeatability of a measurement.

### Instrument

The reference instrument—a questionnaire for the labeling of the elicited emotion after viewing an audiovisual stimulus (see Supplementary Material)—was elaborated by the UC3M4Safety research team for the creation of an audiovisual database (Blanco-Ruiz et al., 2021a,b) and its future use to build an emotional response database capable of measuring physical (voice audio) and physiological variables (heart rate, skin temperature and conductivity, electromyogram, and breathing). The labelling questionnaire of elicited emotions *via* audiovisual stimuli consisted of a brief introduction in which the usage, the way to answer the items, the definition on the scale, and the aim of the study among others were explained. Subsequently, various sets of questions were asked about emotional response and the 12 pre-tagged audiovisual stimuli with the 12 basic emotions (Supplementary Material) were displayed to participants.

The list of emotions for this study (Table 3) was obtained from the coincidences in the Ekman studies (Ekman, 1992, 1999; Ekman and Cordaro, 2011), Izard (2016), Mauss and Robinson (2009), and Plutchik (2001), taking into account the variables used in previous audiovisual databases, such as FilmStim (Schaefer et al., 2010), MANHOB (Soleymani et al., 2012), DEAP (Koelstra et al., 2012), and Emotional Film for Asian culture (Deng et al., 2017), while incorporating the contributions from Ekman (1999, 2016) and the work of Robinson (2008) among others, in which any emotion can be represented in a positive/constructive or negative/destructive way.

### Statistical analysis

The statistical analysis of data was conducted using RStudio® (RStudio, Boston, MA, United States). First, within the scope of calculating the content validity made by expert judges, Aiken’s V test (Penfield and Giacobbi, 2004; Ortega-Toro et al., 2008) was used. Afterwards, in order to know the reliability of the categorical variables (discrete emotions), Kappa coefficient of Fleiss (1971) was calculated following the reference values from Altman (1991). It was an adaptation of Cohen’s Kappa for evaluating the level of agreement between two or more raters. It can be expressed as follows: kappa(κ) = (Po-Pe)/(1-Pe), where Po is the observed agreement and Pe is the expected agreement.

For the continuous variables (PAD indicators), the ICC (Conroy and Metzler, 2003; Correa-Rojas, 2021) was calculated. R functions kappam.fleiss and icc from irr package were used.

## Results

### Expert judges: Content validity of the SAMs and PAD reference values

The quantitative assessment performed by the expert judges provided data about the validity of the content and the shape of Lang’s SAM model, which signalled an Aiken’s V of 0.85 in the best case (Table 1). Aiken’s V values that were similar or greater than 0.8 were found both in the content of valence (0.830), arousal (0.873), and dominance (0.867). However, in terms of shape, only arousal (0.873) was higher than 0.8. Valence (0.722) and dominance (0.643) did not cross this threshold. These results showed a low assessment of the initial information.

The qualitative analysis (Table 2) provided by the expert judges contributed relevant information about the design of a new version of the SAMs: SAM UC3M4Safety.

After analysing the assessments, it was concluded that the gender biases were present in Lang’s SAMs, especially in the case of dominance (the degree of control over the emotional reaction to a stimulus), alluding to the fact that the representation was very masculine, and the lines and expressions were dominant, which can be detrimental when working in emotional identification with a gender perspective.

After this result, the design of the SAMs was reviewed following the experts’ guidelines, creating a seemingly more neutral model (Figure 2), and the terms used in the instructions given to the participants were also reviewed. Afterwards, the expert judges were asked once again to quantitatively assess the items that integrated the instrument, including their degree of relevance and that of precision and adequacy, as well as a global assessment of the instrument itself. The outcomes of the items related to UC3M4Safety’s SAMs demonstrated a high assessment of the final information (Table 1).

**Figure 3:**
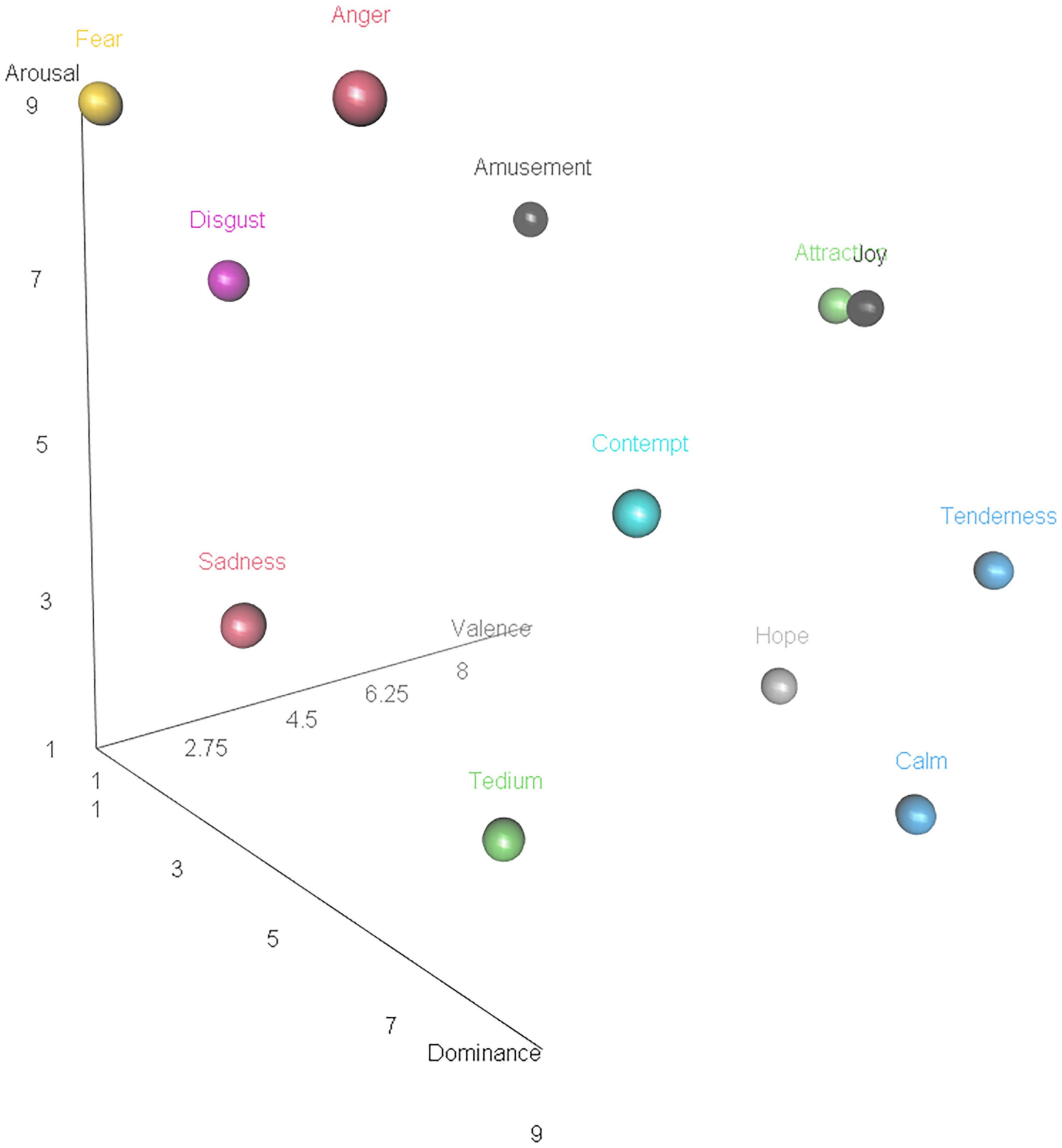
Representation in the pleasure–arousal–dominance space of the reference values established by the expert judges (gold standard). The colours are just to help to identify which point represents each emotion. This representation presents the gold standard in the three-dimensional pleasure–arousal–dominance (PAD) space and places each emotion in a low-medium-high level of excitement, pleasure, and dominance.

In order to establish the reference values (Table 4; Figure 3) that allow the comparisons with the outcomes of the participants, the expert judges were asked to deliver the reference values for the valence, arousal, and dominance variables for each of the 12 basic emotions (Table 3) that represented the 12 basic audiovisual stimuli chosen from the UC3M4Safety audiovisual database (Blanco-Ruiz et al., 2021a,b). In Figure 3, the gold standard representation of these 12 emotions is presented in three-dimensional PAD space, which places every emotion in a low-medium-high level of excitement, pleasure, and dominance.

**Table 4:** Reference values established by the expert judges (Likert 1–9).

**Emotion**	**Mean valence (standard deviation)**	**Mean arousal (standard deviation)**	**Mean dominance (standard deviation)**
Tedium	3.00 (0.00)	1.07 (0.25)	6.23 (0.90)
Joy	8.00 (0.00)	7.00 (0.00)	6.97 (0.18)
Disgust	1.93 (0.25)	7.07 (0.25)	2.47 (0.51)
Attraction	8.00 (0.00)	7.00 (0.00)	6.53 (0.51)
Contempt	3.13 (0.35)	5.13 (0.51)	7.73 (0.69)
Hope	7.00 (0.00)	2.00 (0.00)	6.87 (0.51)
Tenderness	8.20 (0.41)	3.87 (0.51)	9.00 (0.00)
Anger	1.07 (0.25)	8.93 (0.25)	6.13 (1.17)
Fear	1.00 (0.00)	9.00 (0.00)	1.47 (0.51)
Surprise	5.93 (0.25)	7.93 (0.25)	3.00 (0.00)
Calm	6.93 (0.25)	1.00 (0.00)	9.00 (0.00)
Sadness	1.00 (0.00)	3.57 (1.28)	4.40 (1.28)

Mean of the reported values by the experts for the three different dimensions of the PAD space and their standard deviation (between brackets)

### Experiment results

#### Validity and consistency of the discrete-labeling emotions

With the intent of confirming the agreement between the 12 emotions under study (Table 3) that represented the 12 previously tagged audiovisual stimuli (Blanco-Ruiz et al., 2021a,b) and those reported by the participants, a study was conducted using Kappa coefficient of Fleiss (1971). This coefficient measured the degree of correlation among raters of the nominal categories when the same samples were evaluated. The global results showed indices between 0.841 and 0.97 (Table 5) with practically no variation (delta). These results confirmed that the audiovisual stimuli, independent of the assessment system of manikins, generated an emotion in a unique fashion.

**Table 5:** Fleiss’ Kappa index for the measurement of consistency of experienced discrete emotions with both Self-Assessment Manikin models.

**Emotion**	**Global**			**Women**			**Men**		
	**Lang** **SAM**	**UC3M4Safety SAM**	**Delta**	**Lang** **SAM**	**UC3M4Safety SAM**	**Delta**	**Lang** **SAM**	**UC3M4Safety SAM**	**Delta**
Tedium	0.841	0.828	−0.013	0.739	0.724	−0.015	0.983	0.983	0.000
Joy	0.911	0.911	0.000	0.845	0.845	0.000	0.992	0.992	0.000
Disgust	0.892	0.886	−0.006	0.820	0.811	−0.009	0.992	0.992	0.000
Attraction	0.870	0.893	0.023	0.768	0.809	0.041	0.992	0.992	0.000
Contempt	0.878	0.909	0.031	0.784	0.832	0.048	0.992	1.000	0.008
Hope	0.939	0.919	−0.020	0.893	0.858	−0.035	0.992	0.992	0.000
Tenderness	0.924	0.924	0.000	0.868	0.869	0.001	0.992	0.992	0.000
Anger	0.908	0.916	0.008	0.831	0.844	0.013	1.000	1.000	0.000
Fear	0.945	0.945	0.000	0.903	0.903	0.000	1.000	1.000	0.000
Surprise	0.860	0.875	0.015	0.744	0.769	0.025	1.000	1.000	0.000
Calm	0.872	0.900	0.028	0.793	0.839	0.046	0.976	0.976	0.000
Sadness	0.970	0.966	−0.004	0.951	0.938	−0.013	0.992	1.000	0.008

Fleiss’ Kappa coefficient ranges from − 1 to + 1. Negative values represent that the agreement is lower than the expected by chance. On the other hand, positive values imply the rater agreement exceeds chance agreement. Within the positive range, values above 0.80, values between 0.40 and 0.80, and values below 0.40 represent excellent, fair and poor agreement, respectively.

From a gender perspective, we observed that men obtained results with almost no variation (delta) and sustained Kappa index values between 0.97 and 1, that is, they showed practically perfect agreement. Women obtained a Kappa index higher than 0.7, which is a good level of agreement. However, this result confirmed that women have greater variability than men. An improvement was observed in the discrete labelling for women and, to a lesser extent, for men as well when the UC3M4Safety SAMs were used in the questionnaires to classify the experienced emotions.

#### Validity and consistency of emotions of the continuous labeling (pleasure–arousal–dominance)

Once the existence of a high level of agreement between the participants when labelling using discrete emotions was confirmed, the consistency of the continuous tags used for every emotion by the participants was analysed. This analysis considered intraclass and interassessor consistency, that is, if there was a variation in the measurements made by the instrument about the same topic in the same conditions. For this purpose, the ICC was used with the single-rating, absolute-agreement, Two-Way Mixed Effects Model (Table 6). The results corroborated the changes that were taking place in the continuous labelling (PAD) from Lang’s model to UC3M4Safety’s model.

**Table 6:** Assessment of the intraclass pleasure–arousal–dominance for each emotion with both Self-Assessment-Manikin models.

**Emotion**	**Global**			**Women**			**Men**		
	**ICC Lang** **SAM**	**ICC** **UC3M4Safety** **SAM**	**Delta**	**ICC Lang** **SAM**	**ICC** **UC3M4Safety** **SAM**	**Delta**	**ICC Lang** **SAM**	**ICC** **UC3M4Safety** **SAM**	**Delta**
Tedium	0.8675	0.9628	0.095	0.8531	0.9897	0.137	0.8891	0.9359	0.047
Joy	0.5790	0.6700	0.091	0.7803	0.7513	−0.029	0.3626	0.6233	0.261
Disgust	0.8081	0.9356	0.127	0.7455	0.9242	0.179	0.8971	0.9612	0.064
Attraction	0.3188	0.8195	0.501	0.5889	0.9838	0.395	0.5336	0.8349	0.301
Contempt	0.8721	0.9701	0.098	0.7887	0.9952	0.206	0.9831	0.9840	0.001
Hope	0.8066	0.9752	0.169	0.7922	0.9977	0.205	0.8396	0.9531	0.113
Tenderness	0.8246	1.0000	0.175	0.7221	1.0000	0.278	0.9561	1.0000	0.044
Anger	0.9126	0.9685	0.056	0.9212	0.9758	0.055	0.9575	0.9880	0.03
Fear	0.9380	0.9896	0.052	0.8940	0.9936	0.100	0.9882	0.9932	0.005
Surprise	0.7603	0.9561	0.196	0.8043	0.9867	0.182	0.7199	0.9254	0.205
Calm	0.9507	0.9899	0.039	0.9187	0.9914	0.073	0.9862	0.9884	0.002
Sadness	0.6372	0.8526	0.215	0.5527	0.9850	0.432	0.7429	0.9225	0.180
Mean	0.7729	0.9242	0.151	0.7802	0.9645	0.184	0.8213	0.9258	0.104

ICC with the single-rating, absolute-agreement, Two-Way Mixed Effects Model. Values range from 0 to 1. Below 0.50, between 0.50 and 0.75, between 0.75 and 0.90, and above 0.90, the correlation is considered poor, moderate, good, and excellent, respectively. Delta variable is the difference between ICC UC3M4Safety SAM and ICC Lang SAM. The results are disaggregated by gender.

Afterwards, for every emotion provided by the expert judges, agreement with the reference test (golden test) was evaluated (Table 7) in an independent manner for every participant (Figure 7), utilising the ICC index with the single-rating, absolute-agreement, Two-Way Random-Effects Model for each of the labelling methods. The results showed an increase in consistency and agreement between the data corresponding to UC3M4Safety’s SAMs, increasing the ICC to 0.21, 0.22, or 0.23 in the emotions of joy, attraction and surprise, respectively. Additionally, due to that greater agreement, it could be observed that the position of the emotions in the PAD space was more closely adjusted to the one reported by the expert judges, and had a lower standard deviation.

**Table 7:** Degree of agreement between the continuous labelling comparison of the participants with the gold standard for each of the emotions.

**Emotion**	**ICC**		**Model**	**Mean valence (standard deviation)**	**Mean arousal (standard deviation)**	**Mean dominance (standard deviation)**
	**Lang** **SAM**	**UC3M4Safety** **SAM**				
**Tedium**	0.912	0.979	Ref.	3.00 (0.00)	1.07 (0.25)	6.23 (0.90)
			Lang SAM	3.62 (1.00)	1.31 (1.01)	6.88 (1.47)
			UC3M4Safety SAM	3.05 (0.32)	1.01 (0.10)	6.26 (0.85)
**Joy**	0.650	0.861	Ref.	8.00 (0.00)	7.00 (0.00)	6.97 (0.18)
			Lang SAM	8.28 (0.52)	7.31 (1.03)	6.01 (1.46)
			UC3M4Safety SAM	7.99 (0.56)	7.03 (0.38)	6.71 (0.76)
**Disgust**	0.869	0.958	Ref.	1.93 (0.25)	7.07 (0.25)	2.47 (0.51)
			Lang SAM	2.48 (1.19)	7.02 (1.01)	3.26 (1.81)
			UC3M4Safety SAM	2.15 (0.66)	7.06 (0.54)	2.74 (1.11)
**Attraction**	0.656	0.873	Ref.	8.00 (0.00)	7.00 (0.00)	6.53 (0.51)
			Lang SAM	7.50 (0.83)	6.82 (0.57)	6.41 (1.01)
			UC3M4Safety SAM	7.97 (0.18)	6.98 (0.14)	6.53 (0.57)
**Contempt**	0.929	0.984	Ref.	3.13 (0.35)	5.13 (0.51)	7.73 (0.69)
			Lang SAM	3.46 (0.83)	4.56 (1.20)	7.93 (0.78)
			UC3M4Safety SAM	3.05 (0.27)	5.01 (0.12)	7.58 (0.63)
**Hope**	0.860	0.981	Ref.	7.00 (0.00)	2.00 (0.00)	6.87 (0.51)
			Lang SAM	7.51 (0.82)	2.88 (1.65)	6.83 (1.05)
			UC3M4Safety SAM	7.06 (0.24)	2.14 (0.68)	6.99 (0.29)
**Tenderness**	0.894	0.999	Ref.	8.20 (0.41)	3.87 (0.51)	9.00 (0.00)
			Lang SAM	8.14 (0.59)	3.64 (1.22)	8.28 (1.65)
			UC3M4Safety SAM	8.00 (0.00)	4.00 (0.00)	9.00 (0.00)
**Anger**	0.946	0.982	Ref.	1.07 (0.25)	8.93 (0.25)	6.13 (1.17)
			Lang SAM	1.34 (0.70)	8.49 (0.90)	6.05 (1.62)
			UC3M4Safety SAM	1.11 (0.32)	8.83 (0.52)	6.31 (1.09)
**Anger**	0.947	0.992	Ref.	1.00 (0.00)	9.00 (0.00)	1.47 (0.51)
			Lang SAM	1.29 (0.63)	8.54 (0.96)	2.14 (1.44)
			UC3M4Safety SAM	1.09 (0.31)	8.93 (0.32)	1.50 (0.67)
**Surprise**	0.726	0.960	Ref.	5.93 (0.25)	7.93 (0.25)	3.00 (0.00)
			Lang SAM	6.21 (0.80)	7.77 (1.12)	3.56 (1.73)
			UC3M4Safety SAM	5.96 (0.52)	7.93 (0.47)	3.10 (0.63)
**Calm**	0.965	0.994	Ref.	6.93 (0.25)	1.00 (0.00)	9.00 (0.00)
			Lang SAM	6.72 (1.23)	1.30 (0.85)	8.66 (0.93)
			UC3M4Safety SAM	7.05 (0.59)	1.06 (0.37)	8.95 (0.31)
**Sadness**	0.830	0.893	Ref.	1.00 (0.00)	3.57 (1.28)	4.40 (1.28)
			Lang SAM	1.16 (0.66)	3.90 (1.65)	4.09 (1.20)
			UC3M4Safety SAM	1.01 (0.08)	3.05 (0.35)	3.80 (0.98)

ICC with the single-rating, absolute-agreement, and Two-Way Random-Effects Model for each of the labelling methods. Values range from 0 to 1. Below 0.50, between 0.50 and 0.75, between 0.75 and 0.90, and above 0.90, the correlation is considered poor, moderate, good, and excellent, respectively. Mean values for the three different dimensions of the PAD space and their standard deviation (between brackets). Reference (ref.) model corresponds to the values reported by the experts. Lang SAM refers to the values reported using Lang’s manikin’s questionnaire and UC3M4Safety’s SAM as the redesigned one.

**Figure 4:**
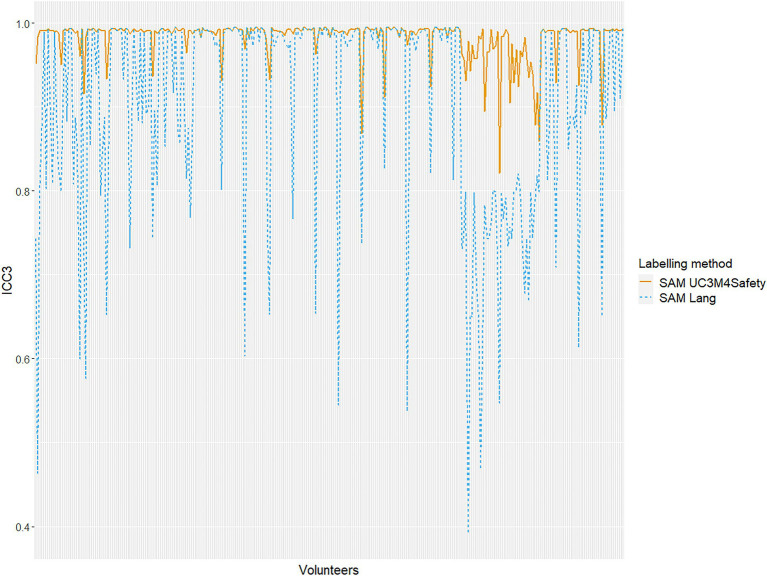
Mean intraclass correlation index of the 12 emotions for each of the participants in relation to the reference test for both models. The *y*-axis represents the mean intraclass correlation coefficient (ICC) value for the 12 emotions with respect to the gold standard. The *x*-axis represents each of the volunteers by identifier. The yellow line shows the results corresponding to answers collected using the UC3M4Safety SAM labelling questionnaire. On the other hand, the blue dotted line presents the values obtained by means of the Lang SAM questionnaire.

Finally, the greater agreement found for UC3M4Safety’s SAMs was studied. In order to do this, the data reported with UC3M4Safety’s SAMs and Lang’s SAMs were analysed, comparing them to the golden labels provided by the expert judges in an individual way for every participant.

Women started off with worse data with Lang’s SAMs to obtaining better results than men according to UC3M4Safety’s SAMs. In Figure 4, the mean correlation index of the 12 emotions for each of the participants in relation to the reference test for both models, as can be observed in almost all cases as a dotted yellow line, is above the blue one, meaning the agreement between the gold standard set by the experts and the participants is higher using the new methodology. Moreover, these results show that there was a greater consistency in the data in relation to the reference (golden) test when the UC3M4Safety SAMs were used, especially in the case of women. Out of 57 participants that obtained the same ICC results with both manikins, only six were women.

## Discussion

This research started from the hypothesis that the tools traditionally used to measure emotions, and therefore train intelligent systems used in affective computing, were not gender neutral. In particular, whether the SAM instrument as a methodology could be considered a neutral tool was evaluated.

The results have shown that the manikins (SAMs), despite being designed with the objective of being neutral, are not perceived as such by the participants. In particular, the case of the graphic representation of dominance is paradigmatic since what is understood as neutral is perceived as a masculine trait. This particular result is not isolated but is part of a mainstream in scientific knowledge and technology that takes the androcentric point of view as neutral (Leavy, 2018). As Haslanger (2000) points out, in science and innovation, men are the norm and women are deviations from it.

The United Nations Organisations (ONU Mujeres, 2021, para. 3) define gender perspective as ‘the assessment process of the consequences for women and men of any planned activity, including laws, policies or programs, in all sectors and at all levels’. The European Commission—the Directorate-General for Research and Innovation—and currently the State Research Agency (Agencia Estatal de Investigación) in Spain argue that engaging the gender research dimension ‘implies that gender is considered a key analytical and explanatory variable in research’ (Dirección General de Investigación e Innovación, 2011, p. 10). This study corroborates the importance of applying the gender perspective so that results are not partial and constitute quality, egalitarian research.

Technology development is increasingly influencing the behaviour of people in everyday life. However, according to Leavy (2018) and Wajcman (2006), the over-representation of men in the design of these technologies could perpetuate gender inequality. Different researchers have demonstrated that AI algorithms are not neutral and contribute to reproducing existing biases in today’s society, the most evident being those of gender and race (O’Neil, 2016; Buolamwini and Gebru, 2018; Noble, 2018; Cirillo et al., 2020). The main types of biases in AI include gender, ethnicity, and age, and these can increase social inequalities or discrimination. Furthermore, these biases affect all sectors in which AI intervenes—from resource allocation in healthcare, justice, education, or employment—and concern both sectors that may look anecdotal—and are not in any way—and relational machines (especially with personal assistants) or vehicles with integrated voice recognition systems (Nurock, 2020).

A clear example is the controversial area of the application of AI in facial recognition software used by law enforcement agencies (Domingo, 2021). Buolamwini and Gebru (2018) proved that the software utilised by the police in the United States had an error rate regarding gender, ethnicity, and age. This error rate clearly favoured young, white men, while negatively affecting black, elderly women.

The newest line in the measurement of emotions for the prediction of scenarios and human behaviour allows interdisciplinary work between disciplines, such as social sciences and engineering, with the aim of making new technologies increasingly “more human.” The applicability of this interdisciplinary synergy that is being applied intends to improve scientific knowledge by introducing the gender perspective into the design of technologies and into the selection of data to train algorithms (Sainz-de-Baranda et al., 2021a, 2022).

The incorporation of areas such as communication with gender perspective in the processes of research of technology and AI allows the advancement of technological development towards solutions that really improve people’s lives (Rituerto-González et al., 2019, 2020; Sainz-de-Baranda et al., 2021a, 2022; Miranda et al., 2022).

Audiovisual communication is greatly contributing to the emerging research field of affective computing. Within immersive virtual reality environments, the elicitation of emotions *via* audiovisual stimuli is showing very intense emotional reactions that can be assimilated into real ones in terms of physical and physiological bio-signals (Blanco-Ruiz et al., 2020; Miranda et al., 2021). However, in order to guarantee a high-quality emotional recognition, the AI system must be trained with adequate data sets, including not only those collected by smart sensors but also the tags related to the elicited emotion. Currently, there are very few techniques available to label emotions. Among them, the SAM, which was created by Lang (1980) and Hodes et al. (1985), is one of the most popular.

The results of this study show that the fact that gender socialisation grants differentiating roles to men and women is not considered. These roles start in childhood, from their initiation in social and cultural life, and are reinforced by the influence of socialising agents. Certain cognitive, attitudinal, and behavioural styles are adopted as well as axiological codes and stereotypical morals and rules that follow the social conduct assigned to each gender (Bosch and Ferrer-Pérez, 2002). The trend of identifying people with their peers—or those just like them—(Igartua and Muñiz, 2008; Soto-Sanfiel et al., 2010) has added to the learning of emotions according to individual experiences, which can serve as an explanation for the existing discrepancy in the discrete labelling between men and women. Men have obtained more favourable results, with a high level of agreement, while women have greater variability. Even though discrete tags are not variable and generally have a high level of agreement with previously reported ones, a raise in the level of agreement when questionnaires containing UC3M4Safety’s SAMs are used has been observed, thus clarifying the new design of manikins when participants experience an emotion during the watching/visualisation of a video after assessing the rest of the PAD characteristics of emotion – especially for women.

In the case of the analysis of emotions reported in a numerical way by the participants and which were represented in a tridimensional fashion in the PAD affective space (valence, activation, and dominance), the differences between the tagged emotion *a priori* and those reported by gender were bigger if both SAM models were applied.

The labelling process of each emotion in the PAD space using the UC3M4Safety SAMs had a higher degree of coincidence with the reference test (gold standard) than that of Lang’s SAMs, both in men and women. These results prove the UC3M4Safety SAM as a reliable and useful tool for the assessment of emotions.

An intersectional feminist approach to new technologies exposes the discriminatory biases of gender, race, and class in the generation and usage of data through information communication technologies (D’Ignazio and Klein, 2020; Blanco-Ruiz, 2022). These results make the inclusion of the gender perspective an imperative in the design of technology and in the generation of databases that are used to train AI systems that coincide with the proposal made by Revi Sterling (2013), who criticises the fact that women, as potential beneficiaries of those technologies, continue to be excluded in design processes.

As pointed out by Schiebinger (2021), identifying gender bias and understanding how it operates is crucially important, “but analysis cannot stop there” (p.3). Future technological developments should be influenced by an intersectional feminist approach (Crenshaw, 1991) in order to avoid reproducing discriminatory gender, race, and class biases, not only in design but also in use (D’Ignazio and Klein, 2020; Blanco-Ruiz, 2022). Incorporating sex, gender, and intersectionality analysis in research is a crucial component that contributes to science and technology (Tannenbaum et al., 2019). Companies such as Google, Amazon, and Facebook are beginning to be aware of the benefits of these inclusive policies. Still, the change must go further; it must permeate the three domains of scientific infrastructure: funding agencies, peer-reviewed journals, and universities (Schiebinger, 2021).

This study is also limited by its own cultural context; it should be tested in other countries to see if the gendered re-reading of the SAM that has been carried out in this study also works in other cultural contexts.

## Conclusion

The new version of UC3M4Safety’s SAMs considers gender perspective in its design and its contribution to the communication field, which allows for the generation of databases that enable better creation of AI systems (affective computing) in order to improve quality of life and avoiding gender biases for both women and men.

The need to revise the procedures used for decades in science—and more concretely, in AI—in order to avoid biases of any kind due to age, ethnicity, gender, or others is left on record.

It has been confirmed that Lang’s SAMs contain gender biases and, consequently, the data resulting from the labelling of emotional reactions that former studies used based on audiovisual databases may be biased, and the generated AI systems could be identifying emotions incorrectly from the analysis of these bio-signals.

This type of research could serve as an inspiration to increase the interest of young people, especially women, in Science, Technology, Engineering, and Mathematics (STEM) fields, as it shows how a small change in the representation of a measuring instrument, such as the SAM, could mean that the perception of half of the population is not considered. Audiovisual and emotions are very attractive areas for young people and can serve as magnets to attract their attention to other possibilities of transferring knowledge to society through the STEM disciplines and their cooperation with other areas of knowledge. The national and international equality policies that foster inclusion of the gender dimension in research and that propel interdisciplinary work—which in our case is that of communication, gender studies, and engineering—produce breakthroughs to develop a more egalitarian scientific knowledge.

## Data availability statement

The datasets presented in this study can be found in online repositories. The names of the repository/repositories and accession number(s) can be found at: https://edatos.consorciomadrono.es/dataverse/empatia.

## Ethics statement

The studies involving human participants were reviewed and approved by Universidad Carlos III de Madrid. The patients/participants provided their written informed consent to participate in this study.

## Author contributions

CS contributed to the study conception and design, did the experiment, performed the material preparation and data analysis, wrote the first draft of the manuscript and commented on previous versions of the manuscript, and read and approved the final manuscript. LG-M performed the material preparation and data analysis, wrote the first draft of the manuscript and commented on previous versions of the manuscript, and read and approved the final manuscript. JM-C and MB-R contributed to the study design, did the experiment, and commented on previous versions of the manuscript. CL-O contributed to the study conception and design, wrote the first draft of the manuscript and commented on previous versions of the manuscript, and read and approved the final manuscript. All authors contributed to the article and approved the submitted version.

## Funding

This work was supported by the Department of Research and Innovation of Madrid Regional Authority under Grant EMPATÍA-CM:Y2018/TCS-5046; and State Research Agency (Spain) under grant PID2019-106695RB-I00/AI-GENBIAS/10.13039/501100011033.

## Conflict of interest

The authors declare that the research was conducted in the absence of any commercial or financial relationships that could be construed as a potential conflict of interest.

## Publisher’s Note

All claims expressed in this article are solely those of the authors and do not necessarily represent those of their affiliated organizations, or those of the publisher, the editors and the reviewers. Any product that may be evaluated in this article, or claim that may be made by its manufacturer, is not guaranteed or endorsed by the publisher.

## Footnotes

^1^Gender refers to the socially defined roles, characteristics and opportunities that are considered appropriate for men, women, boys, girls and people with non-binary identities. Gender is also a product of the relationships between people and can reflect the distribution of power between them (ONU Mujeres, 2021). According to Díaz Martínez, 2016, gender perspective implies that sex and gender are reflected in research content. Gender as a perspective can manifest itself in research questions, theories, approaches, methods and dissemination. This means that sex, gender and their interaction must be adequately represented and addressed in the groups under study, and should be kept in mind if the impact of the research and the results are different. In relation to this, it is interesting to note the work developed by Anne Fausto-Sterling and Londa Schiebinger.

^2^You can access the video clips here: https://edatos.consorciomadrono.es/dataset.xhtml?persistentId=doi:10.21950/LUO1IZ
