# Supplementary material for: Gender biases in the training methods of affective computing: Redesign and validation of the Self-Assessment Manikin in measuring emotions via audiovisual clips
Source: Front Psychol. 2022 Oct 20;13:955530. doi: 10.3389/fpsyg.2022.955530 (PMC9632736; doi:10.3389/fpsyg.2022.955530)
Supplement: Supplementary file 1 [file Data_Sheet_1.docx]

Supplementary Material

# Questionnaire

The set of questions that compose the questionnaire that is repeated every time a stimulus is seen is:

1. In the following question, you will assess the level of arousal or calmness

1 is calmness, relaxation, apathy, drowsiness, lack of activation, and 9 would be chosen if the video has made you feel very agitated, with a high degree of activation, if it has made you feel nervous…, this is represented by the manikin on the right side of your screen. We can also choose intermediate position.


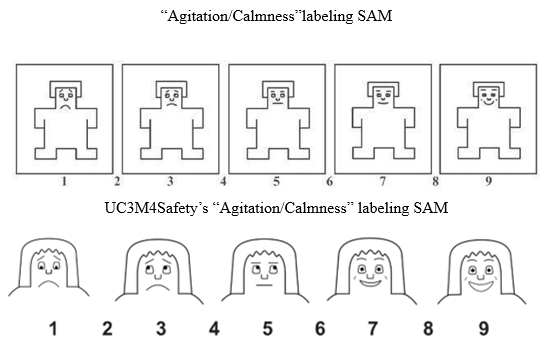


1. In the following question, you will assess the degree of positivity or negativity that the video has made you feel.

You have a scale from 1 to 9 in which 1 means sad, negative, unpleasant, and 9 is happy, positive or pleasant. If you have felt completely positive and satisfied, you would choose the manikin on the right side of your screen (number 9), whereas if you felt negative, sad or angry, you would choose 1, the manikin on the left side of the screen. You can also choose intermediate positions.


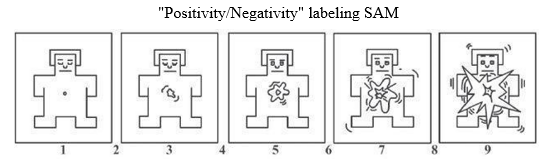


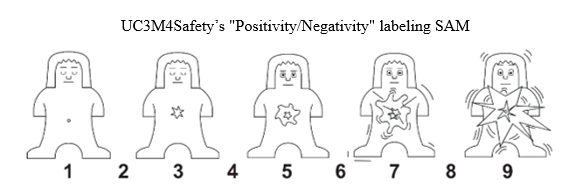


1. In the following question, you will assess the degree of control of the emotion you felt when watching the video.

You have a scale from 1 to 9, where 1 means having very little control, which means that the emotion has taken control of you, and 9 if it is the complete opposite: you had autonomy or a lot of control of the situation. If you have felt very much in control of the emotion, independent, autonomous… you would choose the manikin on the right side of your screen (number 9). On the other hand, if you felt that the emotion took over you or you felt more dependent or heavily influenced by it… we would choose number 1, the manikin on the left side of your screen. You can also choose intermediate positions.


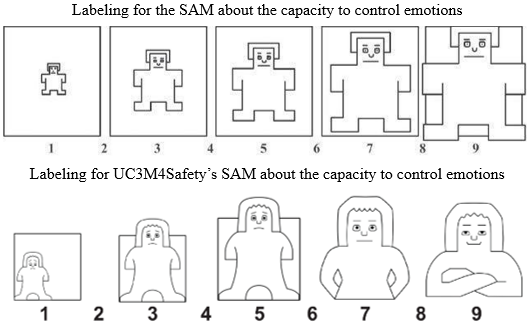


1. Had you ever seen the video?

Yes

No

1. Please, now choose the emotion that better describes what you have felt during the visualization of the video. Choose the emotion—only one—that better describes what you felt during the visualization (the strongest one).

Boredom (weariness, tedium...)

Happiness (joy, satisfaction…)

Disgust (repugnance, aversion…)

Attraction (desire, interest…)

Contempt (indifference, antipathy…)

Hope (trust, safety, faith…)

Gratitude (tenderness, satisfaction…)

Anger (annoyance, ire, irritation, fury, rage…)

Fear (distrust, anguish, anxiety…)

Surprise (amazement, amusement…)

Tranquility (calmness, peace…)

Sadness (distress, sorrow…)

1. What is the intensity of the predominant emotion?

| (Not intense at all) **1** | **2** | **3** | **4** | **5** | **6** | **7** | **8** | **9** | **10** (Very intense) |
| --- | --- | --- | --- | --- | --- | --- | --- | --- | --- |

**
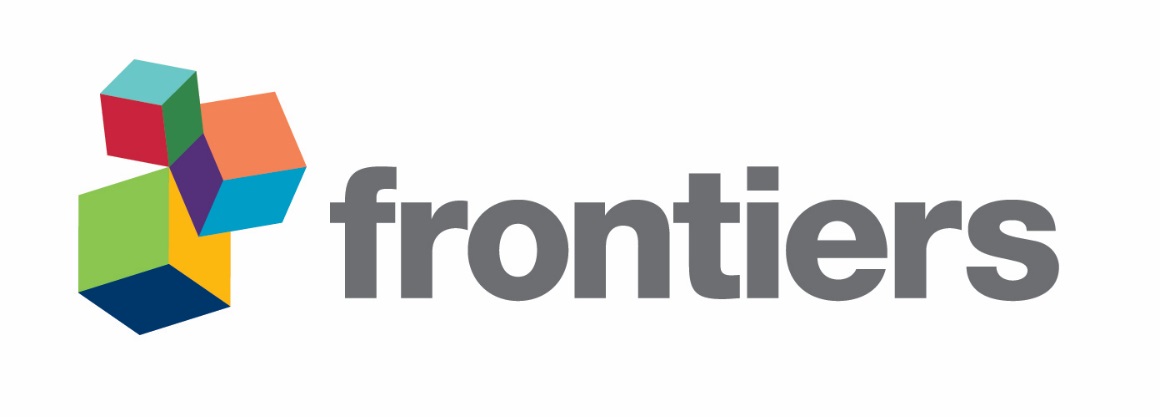
**
